# Matrine in cancer therapy: antitumor mechanisms and nano-delivery strategies

**DOI:** 10.3389/fphar.2025.1656862

**Published:** 2025-09-08

**Authors:** Dandan Meng, Zhenhua Cui, Lexin Li, Qingxin Shang, Xiqi Chen

**Affiliations:** ^1^ College of Traditional Chinese Medicine, Shandong University of Traditional Chinese Medicine, Jinan, Shandong, China; ^2^ Department of Colorectal and anal Surgery, The Second Qilu Hospital of Shandong University, Jinan, Shandong, China; ^3^ Department of Emergency Surgery, Affiliated Hospital of Shandong University of Traditional Chinese Medicine, Jinan, China

**Keywords:** matrine, tumor, nano-delivery system, mechanism, therapy

## Abstract

Cancer remains one of the leading causes of death worldwide. The severe adverse reactions and toxic side effects associated with conventional treatments such as surgery, radiotherapy, and chemotherapy pose significant challenges for researchers and clinical practitioners. These limitations have driven the pursuit of more advanced and effective therapeutic approaches. In recent years, natural products have attracted considerable attention in the field of disease treatment and have become an important source for new drug development. Matrine, a major active component of the traditional medicinal plant *Sophora flavescens*, exhibits a broad range of pharmacological activities, particularly notable antitumor effects. Its antitumor mechanisms include the induction of apoptosis, autophagy, and ferroptosis in tumor cells, as well as the inhibition of tumor cell proliferation, migration, and invasion. With the continuous advancement of therapeutic technologies and the emergence of novel drug delivery strategies, the integration of natural products into cancer therapy has gained renewed significance in the context of innovative delivery systems. Based on this, the present review comprehensively discusses and analyzes the antitumor mechanisms of matrine and its application in nano-delivery systems, highlighting their progress and potential in major disease intervention strategies. This provides new insights for the development and application of advanced drug delivery strategies and technologies in both basic and clinical pharmaceutical research.

## 1 Introduction

Malignant tumors are among the most serious diseases threatening human life and health, with incidence rates steadily rising each year, though their underlying mechanisms remain incompletely understood. According to GLOBOCAN data, by 2040, the global number of new cancer cases is projected to reach 28.4 million, posing a substantial threat to public health (Sung et al., 2021). At present, the main clinical treatment modalities include surgery, chemotherapy, radiotherapy, and molecular targeted therapy. However, their effectiveness is often limited by unsatisfactory therapeutic outcomes and severe adverse effects (Pei et al., 2022; Kessler et al., 2014). In the face of the global challenge of cancer prevention and control, the development of safe and effective antitumor agents has become a major focus for researchers worldwide. In recent years, natural products have demonstrated significant advantages in the comprehensive treatment of cancer, particularly in improving patients’ quality of life, prolonging survival, and reducing the toxic side effects associated with chemotherapy (Yu et al., 2024; Li H. et al., 2024; Lee et al., 2018). They have now emerged as an important strategy in the prevention and treatment of cancer.

Matrine, a major active component of the traditional Chinese medicinal herb *Sophora flavescens Ait.*, belongs to the class of alkaloid compounds (Gao et al., 2024). Studies have demonstrated that matrine possesses a wide range of pharmacological activities, including antitumor, anti-inflammatory, antifibrotic, antidiabetic, antiarrhythmic, antiplatelet, and anti-atherosclerotic effects (Zhang et al., 2020; Zhou et al., 2019; Chen et al., 2022; Huan et al., 2023). Its antitumor mechanisms involve inducing apoptosis and autophagy in tumor cells, inhibiting tumor cell proliferation, invasion, and migration, as well as triggering ferroptosis (Chen et al., 2022). Matrine is characterized by strong pharmacological activity, relatively low toxicity, and significant therapeutic efficacy. It has shown promising therapeutic effects against various acute and chronic inflammatory conditions, cancers, and arrhythmias (Li M. et al., 2024; Halim et al., 2019; Yu et al., 2020; Zhang et al., 2023). Owing to these properties, matrine has been widely studied and applied in the development of traditional Chinese medicine formulations, indicating its broad potential for clinical and pharmaceutical applications.

Malignant tumors currently pose a major global public health challenge, and the innovation of therapeutic strategies has long been a central focus of medical research. However, with the deepening understanding of major diseases such as cancer, inflammation, and neurodegenerative disorders, the limitations of conventional drug delivery methods—such as poor targeting, low bioavailability, and significant toxicity due to poor solubility—have become increasingly apparent (Amiryaghoubi et al., 2023; Qu et al., 2021). To address these issues, nano drug delivery systems (NDDS) have emerged and become a research hotspot in drug development and precision therapy in recent years. Compared with traditional drug delivery methods, targeted NDDS offer several advantages, including improved targeting, enhanced bioavailability, and reduced toxicity (Amiryaghoubi et al., 2023; Qiao et al., 2022; Luo Y. et al., 2023). The rise of NDDS has provided new strategies for the modernization and development of traditional Chinese medicine monomers such as matrine. By encapsulating matrine in liposomes, polymeric nanoparticles, solid lipid nanoparticles (SLNs), nanocrystals, nanogels, or stimulus-responsive nanoplatforms, its solubility and stability can be significantly improved, while enabling controlled release, tumor targeting, and microenvironmental responsiveness. Some studies have also explored the co-encapsulation of matrine with other anticancer drugs in multifunctional nanosystems to achieve combination therapy and synergistic antitumor effects. These approaches have shown considerable promise in overcoming drug resistance and reducing side effects. Therefore, matrine formulations developed using NDDS platforms represent a key strategy for promoting the translational development of this compound from basic research to clinical application.

Therefore, given the broad-spectrum antitumor activity of matrine demonstrated in various tumor models and the rapid advancement of nano-delivery systems, this review aims to systematically summarize the current research progress on nanotechnology-based matrine delivery strategies in cancer therapy. The focus is placed on elucidating the underlying mechanisms by which matrine induces apoptosis, promotes autophagy, and inhibits tumor metastasis. In addition, we highlight the tumor-targeting capabilities and synergistic therapeutic potential of matrine-loaded nanodelivery systems. This review also evaluates the advantages and challenges of these systems in clinical translation, providing new insights for the development and application of novel drug delivery strategies and technologies in both basic and clinical pharmaceutical research.

## 2 Origin and properties of matrine

*Sophora flavescens* is a well-known traditional Chinese medicinal herb that contains a variety of bioactive constituents. Among them, quinolizidine alkaloids are the major phytochemical group, including matrine, oxymatrine, sophocarpine, and sophoridine, which have been extensively investigated for their pharmacological properties. In addition to alkaloids, S. flavescens also contains flavonoids, triterpenoids, and polysaccharides, which contribute to its broad biological activities (Aly et al., 2019). Matrine is a tetracyclic quinolizidine alkaloid primarily found in plants of the Sophora genus within the Fabaceae family, with the highest concentration present in the roots and rhizomes of Sophora flavescens Ait., a traditional Chinese medicinal herb (Gao et al., 2024).

Specifically, matrine and oxymatrine have been identified as the primary anticancer alkaloids, exerting effects through induction of apoptosis, autophagy, ferroptosis, and inhibition of proliferation, invasion, and angiogenesis. Flavonoids like kurarinone also demonstrate anticancer potential by modulating PI3K/Akt and MAPK signaling pathways, while sophoridine has shown efficacy in suppressing tumor growth and metastasis in preclinical models (Cao and He, 2020). These phytoconstituents may act synergistically or via distinct molecular targets, providing a comprehensive pharmacological basis for the anticancer activity of S. flavescens. Modern pharmacological studies have further demonstrated its wide range of biological activities, including anti-inflammatory, antitumor, and antiviral effects.

Chemically, matrine is classified as a quinolizidine alkaloid with the molecular formula C_15_H_24_N_2_O and a molecular weight of 248.36 Da. Its core structure comprises four fused rings and features a lactam moiety, which is believed to be closely related to its diverse biological activities (Sun et al., 2022). Matrine appears as a white crystalline powder with a bitter taste. It is soluble in water, ethanol, and chloroform, and exhibits a melting point of 77 °C–78 °C (Chen S. et al., 2024). Due to its relatively weak basicity, matrine is commonly formulated as hydrochloride or sulfate salts to enhance its stability and bioavailability. In addition, matrine demonstrates favorable thermal and chemical stability under standard storage conditions. Its ultraviolet (UV) absorption spectrum shows a major peak within the range of 220–230 nm, which is frequently utilized for quantitative analysis and quality control.

From the perspective of structure–activity relationship (SAR), modifications at specific sites of the matrine scaffold markedly influence its pharmacological activities. For instance, the introduction of lipophilic or electron-withdrawing substituents at the C-13 or C-14 positions has been shown to enhance its cytotoxic effects against tumor cells. In addition, altering the degree of saturation of the parent ring system may modulate its anti-inflammatory or antiviral activities. These findings indicate that the diverse pharmacological effects of matrine are closely associated with its rigid polycyclic framework and specific functional groups, thereby providing important theoretical insights for structural optimization and the rational design of matrine-derived drugs (Cai et al., 2018; Li et al., 2023). A comprehensive understanding of the source and physicochemical characteristics of matrine not only contributes to elucidating its pharmacological basis but also provides a theoretical foundation for the design of novel drug delivery systems, structural modification, and the investigation of multi-target mechanisms.

## 3 Antitumor mechanisms of matrine

### 3.1 Inhibition of tumor cell proliferation

Cell proliferation, as one of the fundamental biological functions of living cells, is a highly regulated and orderly process (Goodlad, 2017). The uncontrolled proliferation and other malignant behaviors of tumor cells are major contributors to cancer-related mortality (Turajlic and Swanton, 2016; Hanahan and Weinberg, 2000). The *hematological and neurological expressed 1* (HN1) gene is upregulated in various types of cancer, and growing evidence suggests that HN1 contributes to tumor progression in several malignancies (Javed et al., 2023; Liu et al., 2020). Guo et al. (2023) demonstrated *in vitro* that matrine inhibits the proliferation and promotes apoptosis of triple-negative breast cancer (TNBC) cells by downregulating HN1 expression. According to the latest data from the World Health Organization, colorectal cancer (CRC) ranks third among the most common malignant tumors worldwide, with incidence and mortality rates of 9.6% and 9.3%, respectively (Bray et al., 2024). Cheng et al. (2022) treated CRC cells with various concentrations of matrine and found that matrine significantly inhibited CRC cell proliferation. The treatment led to downregulation of miR-10b-5p expression and upregulation of PTEN protein levels, indicating that matrine may suppress CRC cell proliferation via the miR-10b/PTEN signaling pathway. This finding provides a potential molecular mechanism by which matrine may impede the progression of CRC.

The endoplasmic reticulum lipid raft-associated protein 1 (Erlin1), also known as SPFH1, is a membrane protein of the endoplasmic reticulum and a member of the SPFH domain-containing protein family (Browman et al., 2007). Studies have shown that Erlin1 expression is dysregulated across various tumor differentiation stages, indicating its potential as a diagnostic biomarker for cancer (Xi and Zhang, 2018). Ren et al. (2022) investigated the role of matrine in the pathogenesis of CRC and found that matrine induced the upregulation of Erlin1 in CRC cells. Overexpression of Erlin1 promoted the progression of CRC cells, whereas knockdown of Erlin1 effectively suppressed CRC proliferation. These findings suggest that matrine may inhibit CRC progression by modulating Erlin1 expression, identifying Erlin1 as a novel molecular target of matrine. Acute myeloid leukemia (AML) is a common malignant hematologic disorder characterized by the abnormal proliferation and differentiation of immature myeloid progenitor cells in the bone marrow and peripheral blood (Pelcovits and Niroula, 2013). In the United States, more than 20,000 new cases of AML are diagnosed each year (De Kouchkovsky and Abdul-Hay, 2016).

In recent years, numerous non-coding RNAs (ncRNAs), particularly long non-coding RNAs (lncRNAs) and microRNAs (miRNAs), have been recognized as key regulators of cellular physiology and function (Yamamura et al., 2018). Notably, both lncRNAs and miRNAs are involved in the onset and progression of various human cancers and regulate gene expression in cancer cells (Li et al., 2020; Tsagakis et al., 2020). LncRNAs are known to play critical roles in controlling cell proliferation, apoptosis, and differentiation (Chen et al., 2016; Guo et al., 2017). Zhang PP. et al. (2022) found that matrine inhibited cell proliferation and inflammatory cytokine levels, induced apoptosis, and downregulated the expression of LINC01116 while upregulating miR-592. *In vivo* studies showed that matrine suppressed tumor growth by regulating the LINC01116/miR-592 axis and inactivated the JAK/STAT3 signaling pathway in AML cells through this axis. These results indicate that matrine exerts its anti-AML activity by modulating the LINC01116/miR-592 pathway, thereby leading to the inactivation of JAK/STAT3 signaling.

**FIGURE 1:**
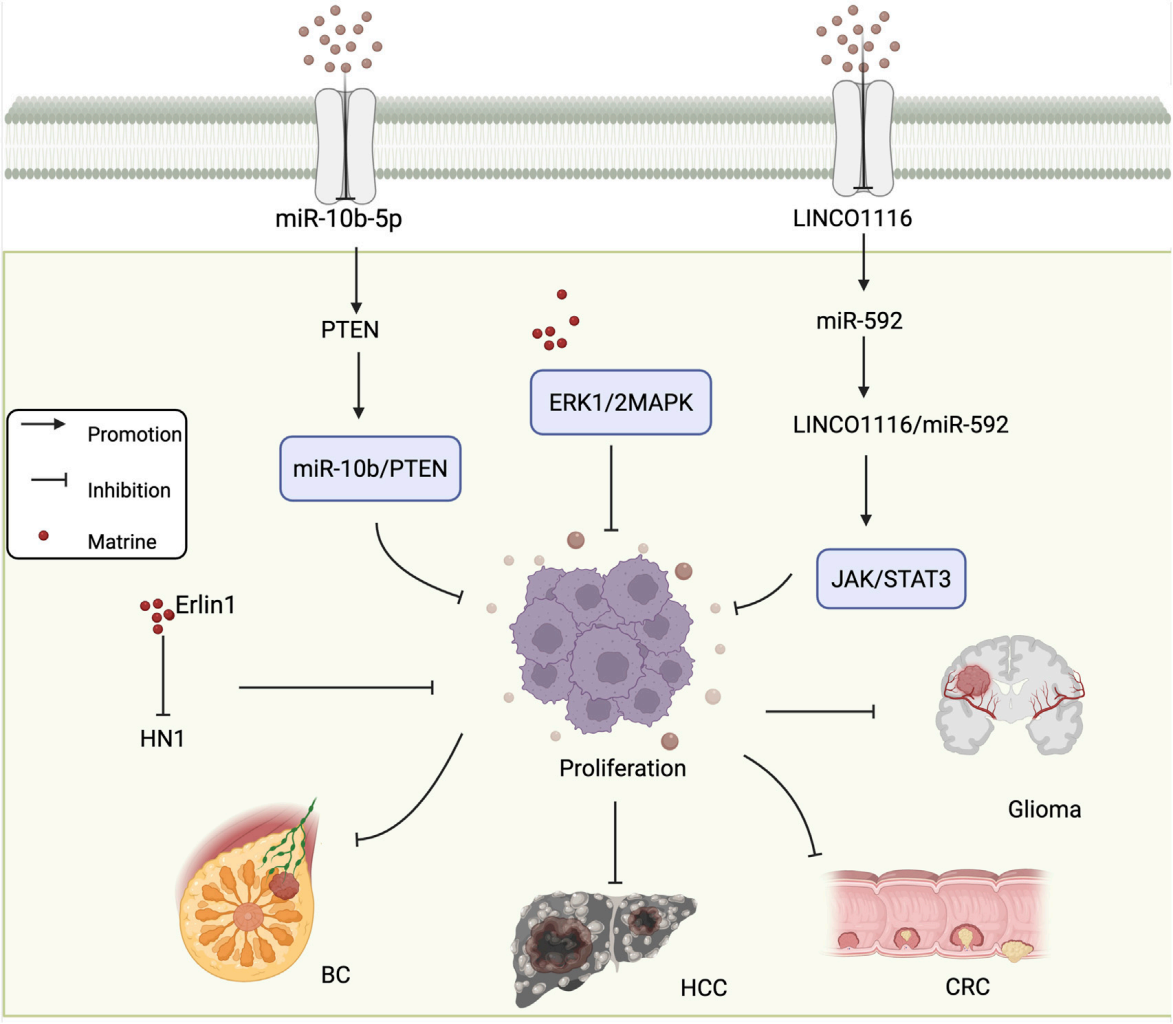
Mechanism of matrine inhibiting tumor cell Proliferation. Matrine regulates the miR-10b/PTEN and ERK1/2 MAPK pathways to suppress proliferation in various cancer types including BC and HCC. It also inhibits HN1 expression via Erlin1. In glioma and CRC, matrine modulates the LINC01116/miR-592 axis and suppresses the JAK/STAT3 signaling pathway. BC, breast cancer; HCC, hepatocellular carcinoma; CRC, colorectal cancer. Arrows indicate activation, blunt lines indicate inhibition, and red dots represent matrine.

Hepatocellular carcinoma (HCC) ranks as the sixth leading cause of cancer-related death worldwide. Its development results from multiple factors, including hepatitis virus infections, consumption of aflatoxin-contaminated food, and liver cirrhosis (Bray et al., 2024; Li and Lan, 2016). One of the key pathogenic features of HCC is its malignant proliferation and invasive capacity, which also represents a major limitation in the clinical efficacy of current anti-HCC therapies (Hu et al., 2019). The ERK1/2 MAPK signaling pathway plays a critical role in regulating tumor cell growth, differentiation, division, and apoptosis, and its abnormal activation has been shown to promote tumor cell proliferation and migration (Zhao et al., 2018). Yu et al. (2020) reported that matrine downregulates the ERK1/2 signaling pathway and inhibits the proliferation and migration of HCC cells in a concentration-dependent manner. These findings suggest that the ERK1/2 pathway may serve as a specific molecular target for matrine in the treatment of HCC. Osteosarcoma is a common and aggressive malignant bone tumor with high incidence and rapid progression. Huang et al. (2024) treated HOS osteosarcoma cells with various concentrations of matrine for 72 h and assessed cell viability using the CCK-8 assay. The results showed that matrine significantly inhibited the viability and proliferation of HOS cells, and this inhibitory effect was enhanced in a dose-dependent manner. These findings indicate that matrine possesses potent anti-proliferative activity against tumor cells and holds great promise for clinical applications in cancer therapy. Figure 1 shows the mechanism of matrine in inhibiting tumor cell proliferation.

### 3.2 Induction of tumor cell apoptosis

Apoptosis, a form of programmed cell death, is a critical physiological mechanism that limits cell population expansion, maintains tissue homeostasis, and eliminates potentially harmful cells (Morana et al., 2022). Thyroid cancer is the most commonly diagnosed endocrine malignancy, with a steadily increasing incidence worldwide (Wiltshire et al., 2016). In papillary thyroid carcinoma (PTC), apoptosis of cancer cells is closely associated with patient prognosis. Fu et al. (2020) found that matrine induces apoptosis in PTC cell lines, decreases the expression level of the anti-apoptotic protein Bcl-2, and activates caspase-3 both *in vitro* and *in vivo*. Furthermore, matrine was shown to inhibit tumor growth *in vivo* via mechanisms partially associated with the downregulation of miR-182-5p. This study elucidated the antitumor mechanism of matrine in PTC and identified miR-182-5p as a potential therapeutic target for matrine-based PTC treatment. TNBC is an aggressive malignancy associated with poor prognosis (Choupani et al., 2023). Guo et al. (2023) demonstrated that matrine significantly inhibited the proliferation, colony formation, and invasion of TNBC cells *in vitro*, while promoting apoptosis. Matrine was found to suppress the expression of HN1. *In vivo* experiments further confirmed that matrine inhibited tumor growth and HN1 protein expression, while upregulating cleaved caspase-3 protein levels. These results suggest that matrine exerts its antitumor effects by suppressing HN1 expression, thereby inhibiting TNBC cell proliferation and promoting apoptosis. Targeting HN1 with matrine may provide novel therapeutic insights for TNBC treatment.

Gliomas, which originate from transformed glial cells, are the most common and lethal primary brain tumors. Characterized by high incidence and mortality rates, gliomas represent a serious global public health concern (Dolecek et al., 2012). Recent studies have identified circular RNAs (circRNAs) as critical regulators involved in gene expression, protein binding, and tumor progression (Guarnerio et al., 2016; You et al., 2015). The PI3K/AKT and Wnt/β-catenin signaling pathways are two fundamental pathways that regulate cell survival, apoptosis, and proliferation (Fresno et al., 2004; Zhang et al., 2013). Chi et al. (2019) observed that matrine induces apoptosis and autophagy in glioma cells by inhibiting the PI3K/AKT and Wnt/β-catenin signaling pathways. This effect is accompanied by the downregulation of circ-104075 and Bcl-9 expression, indicating that matrine exerts its antitumor effects in glioma through the suppression of key oncogenic pathways and circRNA-mediated mechanisms. SHARPIN is a conserved protein of approximately 40 kDa that is widely expressed across various human tissues (Wang et al., 2012).

**FIGURE 2:**
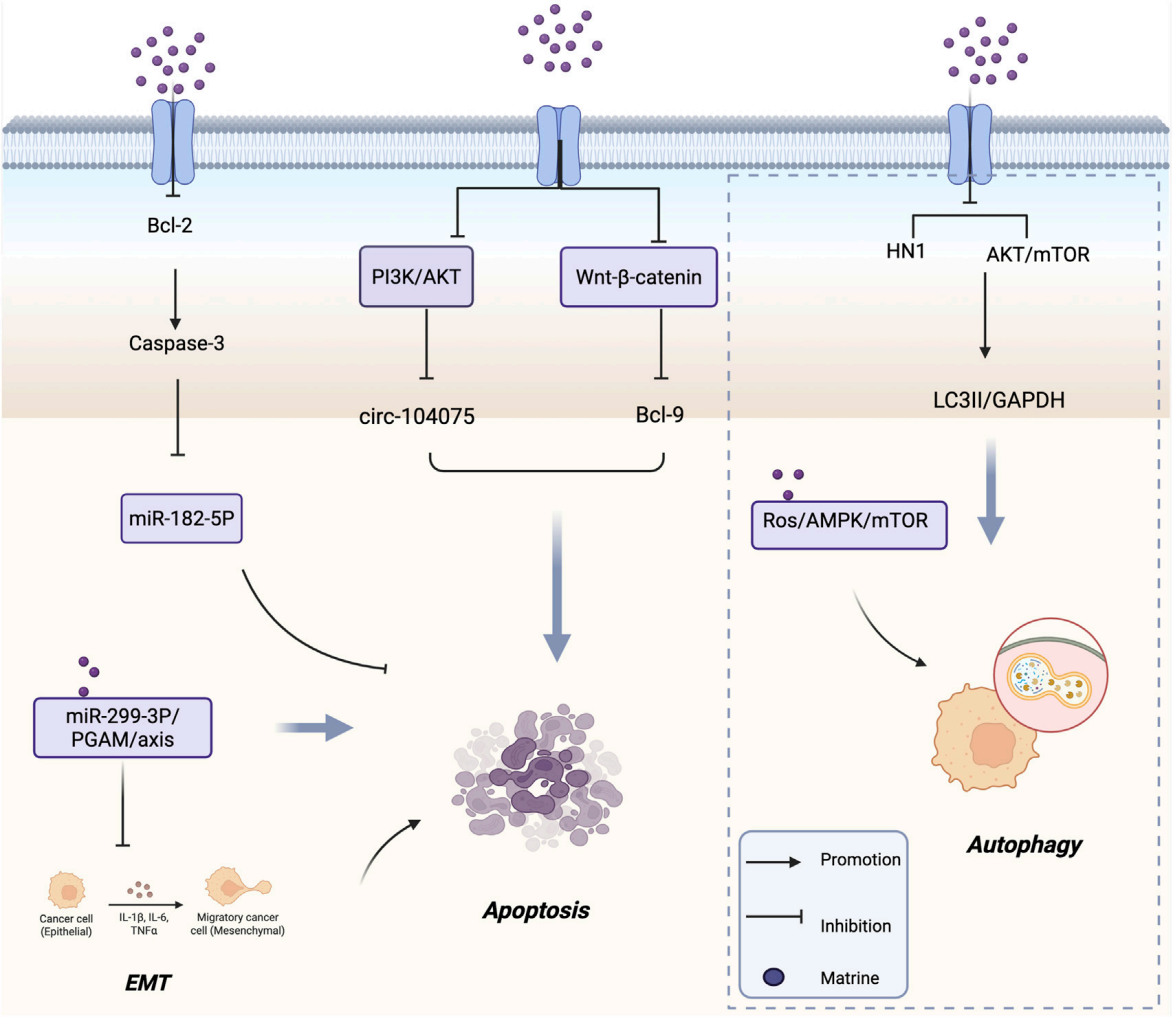
Mechanism of Matrine inducing tumor cell apoptosis and Autophagy. It activates caspase-3 by inhibiting Bcl-2, modulates the PI3K/AKT and Wnt/β-catenin pathways, and regulates circ-104075, Bcl-9, miR-182-5P, and the miR-299-3P/PGAM axis to promote apoptosis and suppress EMT. Meanwhile, matrine triggers autophagy by downregulating HN1 and inhibiting AKT/mTOR signaling via the ROS/AMPK/mTOR pathway. Arrows indicate promotion; blunt lines indicate inhibition; purple dots represent matrine.

Moreover, SHARPIN is found to be amplified in several types of cancer, including prostate, breast, and ovarian cancers (Tian et al., 2019; Zhang et al., 2014; De MELo and Tang, 2015). Zhou et al. (2024), using proteomic analysis and data from The Cancer Genome Atlas (TCGA), discovered that SHARPIN is upregulated in CRC tissues. Furthermore, SHARPIN expression was associated with different TNM stages and poor prognosis. *In vitro* experiments demonstrated that downregulation of SHARPIN induced apoptosis in CRC cells. Treatment with matrine led to decreased SHARPIN expression, which in turn promoted apoptosis and inhibited the proliferation, invasion, and migration of CRC cells. Wang et al. (2022) investigated the role of matrine in regulating HCC through the microRNA (miR)-299-3p/phosphoglycerate mutase 1 (PGAM1) axis. Their study revealed that matrine upregulated miR-299-3p expression *in vitro*, thereby suppressing HCC cell proliferation, invasion, and anti-apoptotic capacity, while also inhibiting epithelial-mesenchymal transition (EMT) and cancer stem cell differentiation. Inhibition of PGAM1 abolished the downregulatory effects of miR-299-3p on HCC cells. These findings suggest that matrine promotes apoptosis and inhibits EMT and stemness in liver cancer cells via the miR-299-3p/PGAM1 axis. Altogether, these results reveal a novel mechanism underlying the antitumor effects of matrine in HCC and identify a potential new therapeutic target for HCC treatment. Figure 2 shows the mechanism of matrine in inducing tumor cell apoptosis.

### 3.3 Induction of autophagy in tumor cells

Autophagy is an evolutionarily conserved stress response and degradation mechanism involving the delivery of damaged or dysfunctional intracellular components to lysosomes via double-membrane autophagosomes for degradation (Kondo et al., 2005; Parzych and Klionsky, 2014). Dysfunctional autophagy has been shown to contribute to tumorigenesis (White et al., 2015). Therefore, inducing autophagy in tumor cells has emerged as an important anticancer strategy. The phosphoinositide 3-kinase (PI3K)-AKT-mammalian target of rapamycin (mTOR) signaling pathway is a well-established negative regulator of autophagy (Xu et al., 2020). Liu et al. (2022) demonstrated that matrine triggers autophagy in neuroblastoma (NB) cells by blocking the AKT-mTOR signaling pathway and inhibiting the phosphorylation of both AKT and mTOR. The PI3K inhibitor 3-methyladenine (3-MA) antagonized the matrine-induced inhibition of cell proliferation, further supporting the notion that matrine’s antitumor activity is at least partially dependent on autophagy. *In vivo*, matrine treatment significantly reduced the phosphorylation of AKT and mTOR in NB xenografts and increased the LC3-II/GAPDH ratio, indicating that matrine functions as an autophagy inducer. Li X. et al. (2024) found that matrine induces apoptosis and cell cycle arrest in multiple myeloma (MM) cells *in vitro*, while simultaneously promoting autophagy. The use of ATG5 siRNA or the autophagy inhibitor spautin-1 partially reversed matrine’s inhibitory effects on MM cells. Conversely, the combination of matrine with the autophagy inducer rapamycin enhanced their antitumor efficacy. These findings suggest that matrine-induced autophagy contributes to MM cell death. Mechanistically, matrine activates the ROS/AMPK/mTOR signaling axis to induce autophagy in MM cells, indicating that combined treatment with matrine and rapamycin may represent a promising therapeutic strategy for MM.

Non-small cell lung cancer (NSCLC) is a leading cause of cancer incidence and mortality worldwide, representing one of the most pressing cancer-related challenges in modern medicine (Mathieu et al., 2022). Luo D. et al. (2023) isolated a novel water-soluble matrine-type alkaloid with anti-NSCLC effects, named sophflarine A (SFA), from the roots of *Sophora flavescens*. Compared to previously identified matrine-type alkaloids, SFA exhibited significantly enhanced antitumor activity *in vitro*. Mechanistically, SFA promoted NSCLC cell death by inducing pyroptosis through activation of the NLRP3/caspase-1/GSDMD signaling pathway. Additionally, it increased reactive oxygen species (ROS) production by inhibiting the PI3K/AKT/mTOR pathway, thereby activating autophagy and suppressing cancer cell proliferation. SFA also inhibited NSCLC cell migration and invasion by blocking the EMT pathway, and further suppressed colony formation and angiogenesis in human umbilical vein endothelial cells. Consistent with these *in vitro* findings, SFA treatment effectively inhibited tumor growth in an orthotopic mouse model implanted with A549 cells. In summary, SFA provides a new theoretical foundation for the clinical application of matrine and exhibits several key properties necessary for its development as a potential therapeutic candidate for NSCLC. Figure 2 shows the mechanism of matrine in inducing tumor cell autophagy.

### 3.4 Inhibition of tumor cell invasion and migration

Tumor cell migration is a major contributor to cancer-related mortality and recurrence, and is closely associated with the loss of cell adhesion induced by EMT activation (Zhang et al., 2021; Akhmetkaliyev et al., 2023). Targeting metastasis is therefore considered one of the most effective strategies to suppress tumor progression (Perlikos et al., 2013). Gallbladder cancer (GBC) is a highly lethal disease and the most common primary invasive tumor of the biliary tract (Kam et al., 2021). Mo et al. (2024) found that matrine significantly inhibited the migration and invasion of GBC cells. By analyzing the mRNA and protein levels of EMT markers and matrix metalloproteinases (MMPs), they further confirmed matrine’s anti-metastatic effect. Compared with the control group, matrine treatment markedly reduced the mRNA levels of Snail, Vimentin, N-cadherin, MMP2, and MMP9, while increasing the expression of E-cadherin. Importantly, matrine also downregulated the protein levels of Vimentin, N-cadherin, MMP2, and MMP9, and upregulated E-cadherin, indicating that matrine inhibits GBC cell invasion and migration at both the transcriptional and translational levels. These findings suggest that matrine exerts its anti-invasive effects by modulating EMT and MMP-related proteins, providing a theoretical basis for its development as a potential therapeutic agent for GBC. Ren et al. (2022), Liu et al. (2020) were the first to identify Erlin1 as a novel target of matrine. Erlin1 is significantly upregulated in tumors; its knockout suppresses CRC cell migration, whereas its overexpression promotes it. Notably, matrine treatment was able to reverse the oncogenic effects of Erlin1 on CRC cell proliferation and migration. When Erlin1 was knocked out, matrine exhibited even more potent antitumor activity in CRC cells. These results suggest that CRC patients with low Erlin1 expression may be more responsive to matrine treatment, positioning matrine as a promising therapeutic strategy for CRC.

Growing evidence indicates that microRNAs (miRNAs) play critical roles in various biological processes, including cell proliferation, invasion, migration, and apoptosis (Yu et al., 2015). In recent years, numerous miRNAs have been identified as tumor suppressors, and their aberrant expression has been linked to oncogenic transformation (Podshivalova et al., 2018; Kwak et al., 2018). Phosphatase and tensin homolog (PTEN) is a well-known tumor suppressor gene with both protein and lipid phosphatase activity (Sansal and Sellers, 2004), and has been reported to inhibit the initiation and progression of multiple cancers. Cheng et al. (2022) found that matrine significantly inhibited CRC cell proliferation, invasion, and migration, downregulated the expression of miR-10b-5p, and upregulated PTEN protein levels. PTEN was identified as a direct target of miR-10b-5p in CRC cells. Both miR-10b-5p knockdown and matrine treatment suppressed cell migration and invasion, while reintroducing PTEN partially reversed these inhibitory effects. These findings suggest that matrine inhibits CRC cell migration and invasion via the miR-10b/PTEN axis, offering a molecular mechanism by which matrine suppresses CRC progression.

**FIGURE 3:**
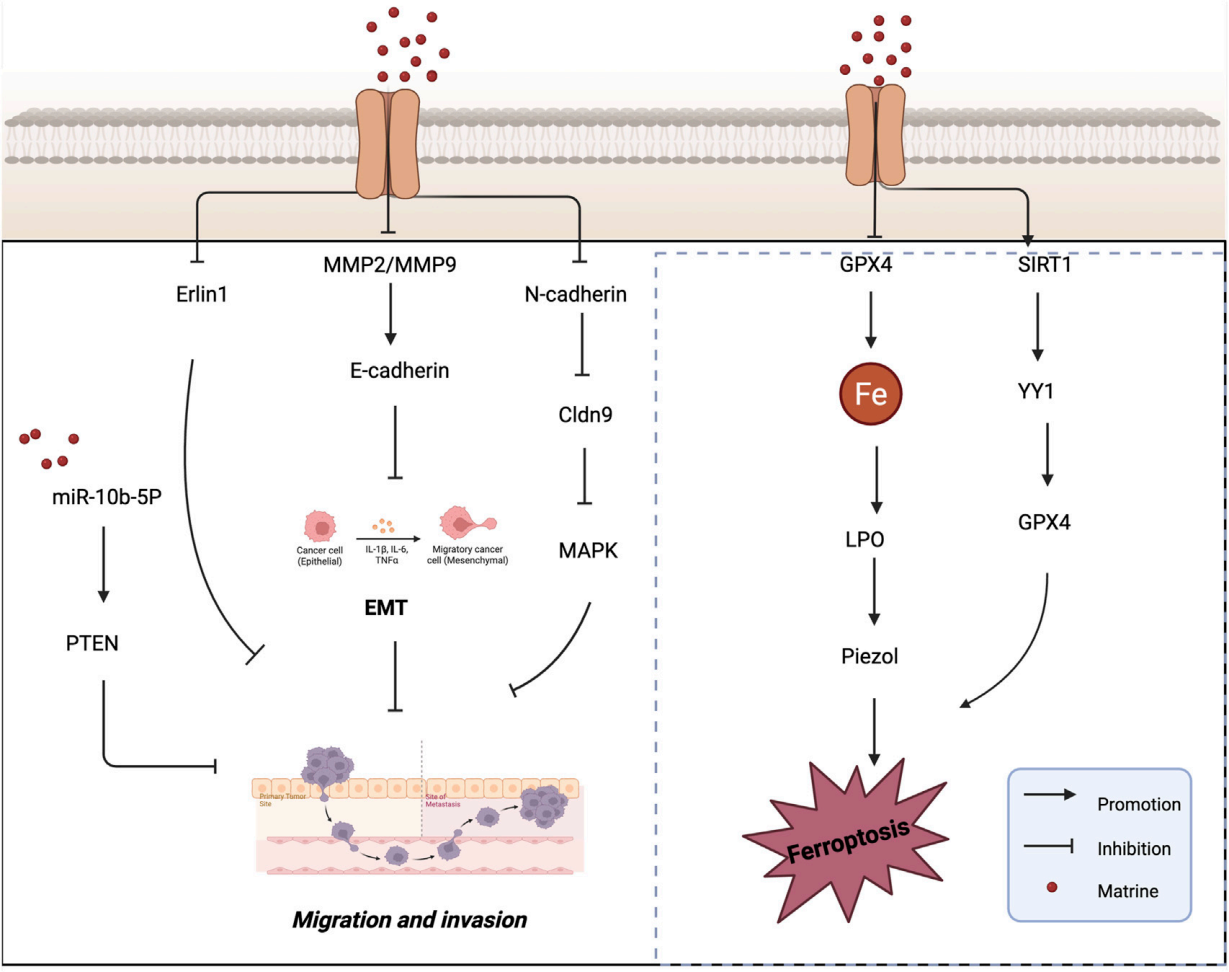
Mechanisms by which matrine inhibits tumor cell migration and invasion and induces ferroptosis. Matrine inhibits tumor cell migration and invasion by regulating EMT-related pathways (e.g., miR-10b-5P/PTEN, MMP2/9, E-cadherin, MAPK) and promotes ferroptosis via suppression of GPX4 and the SIRT1/YY1/GPX4 axis. Arrows indicate activation, blunt lines indicate inhibition, and red dots represent matrine.

Esophageal cancer (EC), a malignant tumor primarily affecting esophageal epithelial cells, is another aggressive cancer type. Xu et al. (2024) investigated the relationship between oxymatrine (OMT) and esophageal squamous cell carcinoma (ESCC). Their study revealed that OMT may inhibit the development and metastasis of ESCC by suppressing the ERK/β-catenin/EMT signaling pathway. *In vivo* studies confirmed that OMT suppressed the growth of ESCC cell-derived tumors in NOG mice without causing damage to other organs. *In vitro*, OMT inhibited ESCC cell migration and invasion by targeting MEK1 (MAP2K1), a key upstream regulator of the ERK pathway, thereby blocking the ERK/β-catenin/EMT axis. Studies have shown that the expression of MMPs is closely associated with the degree of inflammation that promotes tumor cell invasion and migration. Among them, MMP2 and MMP9 play pivotal roles in tumor invasion, metastasis, and angiogenesis (Guo et al., 2021). Du et al. (2023) were the first to report that *claudin-9* (Cldn9) is upregulated in human colon cancer (CC) tissues compared to normal tissues. Silencing Cldn9 in CT26 colon cancer cells significantly inhibited vasculogenic mimicry (VM) formation, as well as cell proliferation, migration, and invasion. This suppression was accompanied by downregulation of N-cadherin, MMP2, and MMP9 expression, along with reduced phosphorylation levels of ERK and JNK, while E-cadherin expression was upregulated. These findings suggest that inhibition of Cldn9 can reverse the EMT process and suppress the MAPK signaling pathway, thereby reducing the proliferation and invasiveness of CC cells. Collectively, this research provides a potential therapeutic candidate and target for VM-based anti-metastatic strategies in colon cancer. Figure 3 shows the mechanism of matrine in inhibiting tumor cell invasion and migration.

### 3.5 Induction of ferroptosis in tumor cells

The regulation of cell death processes is critically important in the development and treatment of cancer. Ferroptosis is a newly identified form of programmed cell death that is morphologically, biochemically, and genetically distinct from apoptosis, pyroptosis, and autophagy (Dixon et al., 2012). Biochemically, ferroptosis is characterized by glutathione (γ-glutamylcysteinylglycine, GSH) depletion and reduced activity of glutathione peroxidase 4 (GPX4). As a result, lipid hydroperoxides are no longer efficiently reduced by GSH under GPX4 catalysis. This leads to the accumulation of lipid peroxides, which are oxidized by ferrous ions (Fe^2+^) in a Fenton-like reaction, generating excessive ROS and ultimately triggering ferroptotic cell death (Zhang C. et al., 2022). The discovery of ferroptosis has garnered significant attention in the scientific community. Numerous studies have demonstrated that tumor cells require more iron than normal, non-cancerous cells (Steve et al., 1994; Torti and Torti, 2013). This iron dependency suggests that cancer cells may be more susceptible to ferroptosis, making it a promising therapeutic target in oncology. Jin et al. (2024) conducted an *in vivo* study and found that matrine effectively inhibited tumor growth in a SiHa cell-induced tumor-bearing mouse model, without causing noticeable harm to the animals. Matrine treatment significantly reduced GPX4 protein levels while increasing lipid peroxidation and intracellular Fe^2+^ content, indicating that matrine induces ferroptosis. Further investigation revealed that matrine markedly upregulated the expression of Piezo1, a mechanosensitive ion channel, while having no significant effect on the expression or interaction of transferrin receptor (Tfr) and system Xc^−^ (xCT). These findings suggest that matrine exerts its antitumor effects in cervical cancer by activating the Piezo1 channel to trigger ferroptosis, thereby offering a novel protective mechanism against tumor progression.

SIRT1 (Silent Information Regulator 1) plays a crucial role in various biological processes and has been shown to deacetylate the transcription factor Yin Yang 1 (YY1) (Du et al., 2021). Additionally, several studies have reported that SIRT1 can promote apoptosis in cancer cells and function as an antitumor factor in cancer progression (Lee et al., 2020). Hu et al. (2025) investigated the mechanism by which OMT affects the development and progression of HCC. They founcd that OMT induced cell death and inhibited proliferation in HCC cells, while simultaneously downregulating the expression of YY1 and GPX4 and upregulating SIRT1 expression. Ferroptosis was identified as the primary mode of OMT-induced cell death. Overexpression of GPX4 or YY1, or inhibition of SIRT1, reversed the ferroptosis induced by OMT, confirming the involvement of this pathway. Furthermore, in an HCC xenograft model, OMT suppressed tumor growth via the SIRT1/YY1/GPX4 signaling axis. These findings suggest that OMT reduces HCC cell viability and induces ferroptosis through regulation of the SIRT1/YY1/GPX4 axis, highlighting a potential novel therapeutic mechanism for the treatment of liver cancer. Figure 3 illustrates the mechanism by which matrine induces ferroptosis in tumor cells, and Table 1 summarizes its antitumor mechanisms.

**TABLE 1:** The antitumor mechanisms of Matrine.

Mechanism	Model/Tissue	Concentration	Duration of administration	Method of extraction	Types of cancers	Ref.
Inhibition proliferation	MDA-MB- 45, HCC- 1806	0, 1.0, 2.0 and 4.0 mM	24, 48, 72h	Pure compound	TNBC	Guo et al. (2023)
	HT29, DLD1	0, 0.4, and 0.8 mg/mL	0–48 h	Pure compound	CRC	Cheng et al. (2022)
	HT-29, RKO	1.722 mg/mL	24 h	Pure compound	CRC	Ren et al. (2022)
	THP-1 and HL-60 cells, HS-5 cells	0.5, 1, or 2 g/L	72 h	Pure compound	AML	Zhang et al. (2022a)
	HepG2	1, 2, 4 mg/mL	24 h	Pure compound	HCC	Yu et al. (2020)
	HOS cells	5 μg/mL	72 h	Pure compound	Osteosarcoma	Huang et al. (2024)
Induction apoptosis	TPC-1, BCPAP, and K1 cell line	0.25, 0.5, 1 mg/mL	24h	Pure compound	PTC	Fu et al. (2020)
	BALB/c athymic nude mice	50 mg/kg	6w	Pure compound	PTC	Fu et al. (2020)
	MDA-MB-453, HCC-1806	0, 1.0, 2.0, 3.0, 4.0 and 5.0 mM	48 h	Pure compound	TNBC	Guo et al. (2023)
	U251 cells	0, 0.1, 0.5, 1, 2, 4 nm	24h	Pure compound	Glioma	Chi et al. (2019)
Induction of autophagy	SK-N-AS , SK-N-DZ	0, 0.2, 0.4, 1, 2, 4, and 6 mM	24, 48 or 72 h	Pure compound	Neuroblastoma	Liu et al. (2022)
	NCI-H929, RPMI8226, and U266	0, 0.5, 1.0, 1.5 mg/mL	12, 24, or 48 h	Pure compound	MM	Li et al. (2024c)
Inhibition of invasion and migration	GBC-SD cell line, NOZ cell line, SGC-996	0, 5, 10umol/L	24h	Pure compound	GBC	Mo et al. (2024)
	HT29 and DLD1	0, 0.4, and 0.8 mg/mL	0–48 h	Pure compound	CRC	Cheng et al. (2022)
	HCT116, SW480, and KM12, CT26	0, 0.25, 0.5, or 1 mM	24, 48h	Pure compound	Colon Cancer	Du et al. (2023)
Induction of ferroptosis	SiHa cells	0.25, 0.5, 1, 2 mg/mL	24 h	Pure compound	Cervical cancer	Jin et al. (2024)
	female CB17 SCID mice	25, 50, 75 mg/kg	22d	Pure compound	Cervical cancer	Jin et al. (2024)
	Hep3B and HepG2	0, 5, 10, 20, 30, 40 μg/mL)f	24h	Pure compound	HCC	Hu et al. (2025)

## 4 Pharmacokinetic advantages of NDDS

NDDS, typically defined as delivery platforms with particle diameters ranging from 10 to 1,000 nanometers, represent a significant advancement over conventional drug delivery methods Compared to traditional systems, NDDS offer enhanced bioavailability and stability for poorly soluble drugs. Moreover, nanoparticles that are specifically designed and surface-modified can improve drug targeting and controlled release, thereby increasing the therapeutic index of the administered agents (Hassan et al., 2017). NDDS mark a major breakthrough in the field of drug delivery systems (DDSs), with the primary objective of delivering the appropriate amount of a therapeutic agent to the right site at the right time. This approach aims to maximize drug utilization, enhance therapeutic efficacy, minimize toxicity, and reduce overall treatment costs (Jain, 2008).

NDDS encompass a broad range of platforms, including micelles, liposomes, polymeric nanoparticles, biologically derived carriers (e.g., exosomes), biomimetic nanocarriers, metal-organic framework nanoparticles, and inorganic nanomaterials. Their effectiveness in drug delivery is attributed to unique properties such as particle size, surface charge, morphology, and material composition, as well as the ability to undergo targeted surface modifications. These systems can be used to efficiently deliver a variety of therapeutic agents, including small molecules and macromolecules such as nucleic acids. One of the key advantages of nanotechnology-based drug delivery is the enhanced permeability and retention (EPR) effect, which allows for preferential accumulation of nanoparticles in tumor tissues. Additionally, surface modification with targeting ligands can further enhance drug distribution within tumors and improve penetration into deeper tumor regions, ultimately leading to superior therapeutic outcomes (Gabizon et al., 2006; Nikpoor et al., 2015). At the same time, NDDS have demonstrated significant advantages in improving the pharmacokinetic behavior of drugs due to their unique physicochemical properties and tunability. From the perspective of the four fundamental pharmacokinetic processes—absorption, distribution, metabolism, and excretion (ADME)—NDDS can optimize drug behavior at multiple stages *in vivo*, thereby enhancing therapeutic efficacy and reducing adverse effects (Hamidi et al., 2013).

### 4.1 Absorption

Conventional drugs often face challenges such as poor solubility, limited membrane permeability, and degradation in the gastrointestinal tract, which severely restrict their bioavailability. NDDS can improve drug solubility and stability, enhance adhesion and mucosal penetration, and thus effectively promote transmembrane absorption. For instance, carriers such as liposomes, solid lipid nanoparticles, and polymeric nanoparticles can encapsulate unstable or poorly soluble drugs, protecting them from degradation by gastric acid and enzymes while improving absorption efficiency in the small intestine (Hamidi et al., 2013).

### 4.2 Distribution

NDDS can be engineered to achieve targeted distribution to specific tissues or cells by adjusting parameters such as particle size, surface charge, and ligand modification. For example, nanoparticles modified with tumor-targeting ligands (e.g., folic acid, RGD peptides) can actively recognize and accumulate in tumor tissues, significantly increasing local drug concentration while minimizing nonspecific exposure to healthy tissues. In addition, NDDS can leverage the enhanced EPR effect to achieve passive targeting, effectively prolonging drug retention at the tumor site (Alexis et al., 2008).

### 4.3 Metabolism

Certain drugs are rapidly metabolized and inactivated by hepatic enzymes due to first-pass metabolism. NDDS can extend drug half-life by encapsulating the drug to evade enzymatic recognition or by utilizing targeted delivery strategies to bypass first-pass metabolism. For instance, PEGylated nanoparticles (modified with polyethylene glycol) can markedly reduce clearance by the reticuloendothelial system (RES), delay drug metabolism, and enhance systemic drug exposure (Mitche et al., 2021).

### 4.4 Excretion

NDDS offer the ability to modulate drug elimination pathways and rates effectively. By adjusting particle size and surface characteristics, NDDS can reduce rapid glomerular filtration and extend plasma half-life. Conversely, for drugs requiring rapid elimination, degradable nanocarriers can be designed to ensure timely clearance after therapy, thereby reducing toxicity risks. Additionally, some carriers can be gradually degraded *in vivo* into non-toxic small molecules, which are safely excreted via hepatobiliary or renal pathways (Hamidi et al., 2013).

In summary, NDDS exhibit clear advantages across all four key pharmacokinetic processes, providing superior control over the *in vivo* fate of drugs. This represents a crucial strategy for improving therapeutic indices and enhancing the clinical prospects of drug candidates.

## 5 Types of matrine-based NDDS

**FIGURE 4:**
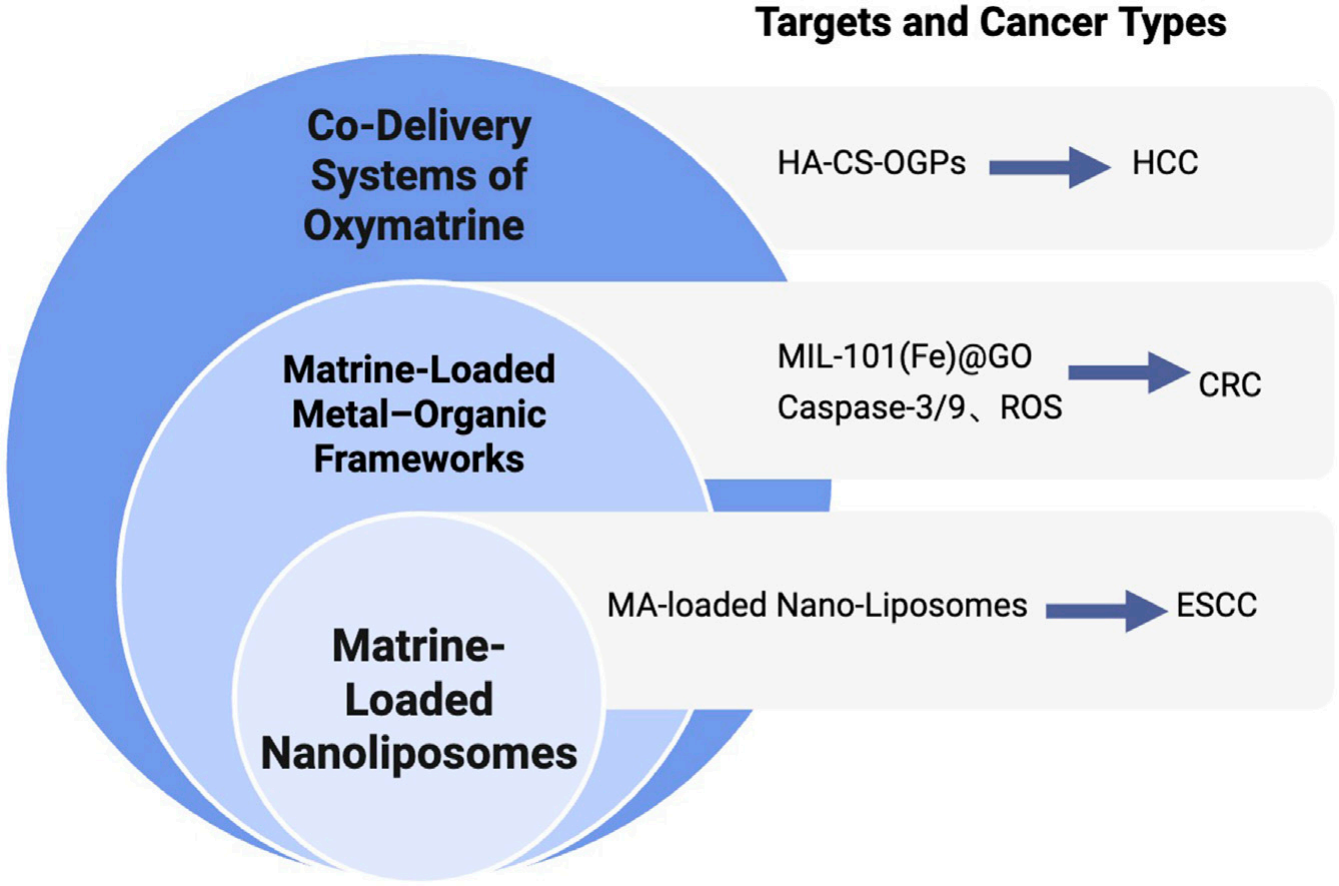
Nano-delivery strategies of matrine for cancer therapy. Overview of NDDS applied for matrine and oxymatrine delivery, including nanoliposomes, metal–organic frameworks (MOFs), and co-delivery systems, with applications in ESCC, CRC, and HCC.

NDDS represent a groundbreaking pharmaceutical technology that emerges from the interdisciplinary integration of theories and methodologies across fields such as physical chemistry, biology, polymer science, materials science, mechanical engineering, and electronics (Liechty et al., 2010). Compared to traditional drug delivery methods, NDDSs offer several notable advantages (Li et al., 2019). NDDSs demonstrate numerous benefits over conventional drug formulations, including enhanced targeted delivery and controlled drug release (Williams et al., 2013), improved solubility and bioavailability (Li et al., 2013), reduced toxicity and side effects, as well as the ability to cross physiological barriers such as the blood–brain barrier (BBB) (Yoo et al., 2010). Moreover, functional or structural modification of nanoparticles can reduce off-target effects, prolong systemic circulation time, and increase delivery efficiency *in vivo* (Maeda et al., 2009). Given these advantages, combining traditional Chinese medicine monomers such as matrine with advanced NDDS platforms has become a promising and emerging trend in the development of anti-tumor therapies. This approach not only enhances the pharmacokinetic profile of the active compound but also provides new opportunities to optimize therapeutic outcomes and overcome the limitations of conventional administration methods. Figure 4 illustrates the types of nano drug delivery systems (NDDS) applied in the delivery of matrine and oxymatrine.

Figure 4 Types of NDDS Applied in the Delivery of Matrine and Oxymatrine. Overview of NDDS applied for matrine and oxymatrine delivery, including nanoliposomes, metal–organic frameworks (MOFs), and co-delivery systems, with applications in ESCC, CRC, and HCC.

### 5.1 Matrine-loaded nanoliposomes

ESCC is typically diagnosed at an advanced stage, necessitating more effective therapeutic strategies. In China, approximately 90% of esophageal cancer cases each year are classified as ESCC (Watanabe et al., 2020). Studies have shown that nano-sized drug carriers can enhance drug absorption and improve therapeutic outcomes (Gabizon et al., 2006). Generally, lipid-based drug formulations with particle sizes ranging from 10 to 100 nanometers can penetrate and accumulate in tumor tissues, while minimizing toxicity in healthy tissues. This is due to the larger vascular fenestrations in tumor tissues—often several hundred nanometers in diameter—compared to those in normal capillaries. To enhance the antitumor activity and bioavailability of matrine, Zhao et al. (2024) developed matrine-loaded nanoliposomes (MNLs). Liposomes are subject to rapid clearance by the reticuloendothelial system (RES); however, they can accumulate in target tissues via the EPR effect. The EPR effect refers to the preferential leakage and retention of macromolecules in tumor tissues due to the unique vasculature of tumors, which facilitates the passive targeting of nanoparticle-based drugs (Maeda et al., 2000; Sawant and Torchilin, 2012).

In their study, Zhao and colleagues utilized a film-ultrasonic method to prepare MNLs and applied them to KYSE-150 ESCC cells. The results demonstrated that MNLs significantly enhanced the anticancer efficacy of matrine in a concentration-dependent manner, as evidenced by reduced cell viability and a lower IC_50_ value. MNLs effectively suppressed cell proliferation and induced apoptosis in KYSE-150 cells. These findings suggest that matrine-loaded nanoliposomes represent a promising and effective strategy for enhancing the anti-ESCC activity of matrine, offering a potential natural therapeutic agent and delivery platform for the clinical treatment of ESCC.

### 5.2 Matrine-loaded metal–organic frameworks

Metal–organic frameworks (MOFs) are a class of porous hybrid materials composed of metal ions or clusters coordinated to organic ligands. These materials possess distinctive characteristics such as high surface area, tunable pore size, ease of synthesis, surface modifiability, and controlled chemical instability (Mckinlay et al., 2010). Due to these properties, MOFs have become a research hotspot as drug delivery platforms for cancer therapy.

Graphene oxide (GO), with its layered structure, offers a favorable template for the *in situ* growth of MOF particles. The combination of MOFs with GO not only enhances drug permeability and retention but also improves drug absorption efficiency. This hybrid system provides more efficient drug-binding sites and enhances the hydrophilicity of the composite materials (Abazari et al., 2022; Hu et al., 2023; Wang et al., 2023). Shen et al. (2024) developed a novel dual-drug delivery system composed of MIL-101(Fe)@GO co-loaded with luteolin and matrine. They successfully synthesized a composite carrier based on NH_2_-MIL-101(Fe) and GO with uniform particle size and stable structure. This system effectively encapsulated both luteolin and matrine, forming a pH-responsive drug delivery platform capable of releasing luteolin under acidic conditions—mimicking the tumor microenvironment. *In vitro* pharmacodynamic studies confirmed the synergistic effect between NH_2_-MIL-101(Fe) and GO, with GO significantly enhancing the anti-colorectal cancer efficacy of the composite system. The platform effectively suppressed the proliferation and migration of RKO CRC cells by increasing ROS production and upregulating the expression of caspase-3 and caspase-9. This research highlights the advantages of combining MOFs with GO and demonstrates the potential of MOF@GO-based delivery systems in drug loading and anticancer applications. Moreover, it provides a novel and promising approach for multi-component therapeutic strategies against CRC.

### 5.3 Co-delivery systems of oxymatrine

Studies have shown that the incidence of multidrug resistance (MDR) in primary liver cancer ranges from 84.6% to 100% (Ceballos et al., 2019). Currently, combination chemotherapy is commonly used in clinical settings to improve therapeutic efficacy. However, its overall effectiveness remains unsatisfactory. Compared with free drugs or those delivered separately via individual nanocarriers, the co-delivery of anticancer agents using a single nanocarrier has been demonstrated to be significantly more effective in reversing MDR both *in vitro* and *in vivo* (Zhang et al., 2016). This co-delivery strategy offers several advantages, including precise and controlled drug release profiles that help maintain optimal drug ratios at target sites (Gurunathan et al., 2018). Proper drug pairing is crucial for successful combination therapy. Co-delivering anticancer drugs with chemosensitizers—agents that modulate MDR mechanisms—has shown better therapeutic performance than simply combining multiple anticancer agents (Zhao et al., 2015).

In this context, OMT, a natural compound with notable antitumor and low-toxicity properties, has emerged as a promising candidate for MDR-targeted co-delivery systems. When loaded into nanocarriers alongside traditional chemotherapeutic agents or MDR modulators, OMT can enhance drug accumulation in tumor tissues, improve chemosensitivity, and reduce systemic toxicity. This approach not only maximizes therapeutic synergy but also provides a new direction for overcoming drug resistance in HCC and other refractory cancers. Chitosan (CS) is a biopolymer known for its excellent biocompatibility and permeability, as well as a variety of biological properties, including antibacterial and anticancer activities (Mikušová and MIkuš, 2021). Hyaluronic acid (HA), a negatively charged linear glycosaminoglycan composed of D-glucuronic acid and N-acetyl-D-glucosamine, has been shown to enhance drug accumulation in cancer cells that overexpress CD44—a non-kinase transmembrane glycoprotein—when used to modify chitosan nanoparticles (Wang et al., 2017; Yang et al., 2015; Suh et al., 2017; Xu et al., 2017). Based on these principles, Chen X. et al. (2024) developed a plant-derived biocompatible nanocarrier system for the co-delivery of OMT and glycyrrhizin (GL) to treat HCC. In this system, OMT and GL were co-loaded into organelle-like plant-derived particles (OGPs) for combination therapy. CS and HA were used to modify the surface of OGPs to control drug release and enable active targeting of tumor cells. The loading ratio of OMT to GL in the nanocarrier was fixed at 1:1.

Further studies on *in vitro* cytotoxicity and cellular uptake using HepG2 cells demonstrated that the plant-based co-delivery system exhibited significant synergistic antitumor activity. This effect was further enhanced by CS and HA modification. These findings suggest that the HA-CS-OGPs platform for co-delivery of OMT and GL is a promising strategy for reversing multidrug resistance in cancer therapy and offers a novel, biocompatible approach to combination treatment in HCC.

In summary, natural products have emerged as a vital resource in the development of anticancer drugs. However, the low lipid solubility of many natural compounds often leads to poor absorption efficiency, significantly limiting their chemotherapeutic potential. In recent years, plant-derived nanocarriers have shown great promise as novel delivery systems. By encapsulating bioactive compounds within phospholipid-based structures, these carriers can markedly improve the absorption characteristics of natural drugs. Although combination therapy has been widely adopted in clinical oncology, the inherent pharmacokinetic properties and biodistribution profiles of anticancer agents often present major barriers to enhanced therapeutic efficacy. Nano-based drug delivery systems offer a compelling solution by co-loading multiple anticancer agents into a single nanocarrier. This approach not only enhances the accumulation and retention of drugs within tumor tissues but also enables precise control over drug release profiles. As a result, it significantly increases the therapeutic index while minimizing systemic side effects.This strategy presents a scientifically grounded and clinically translatable paradigm for cancer therapy, offering new opportunities for improving treatment outcomes through advanced, nanotechnology-enabled drug delivery.

## 6 Conclusion and perspectives

Matrine, as a natural alkaloid derived from Sophora flavescens, exhibits a diverse array of pharmacological activities, among which its antitumor potential has attracted considerable attention. The compound demonstrates multifaceted anticancer mechanisms, including the induction of apoptosis, autophagy, and ferroptosis, as well as the suppression of tumor cell proliferation, migration, and invasion. These characteristics make matrine a promising candidate for antitumor therapy, especially as part of integrative treatment strategies (You et al., 2020).

However, its clinical application is significantly hindered by inherent drawbacks such as poor water solubility, low bioavailability, and lack of tumor-targeting ability. The incorporation of matrine into NDDS offers a viable strategy to address these limitations. NDDS can improve the pharmacokinetic profile of matrine by enhancing its solubility, protecting it from premature degradation, promoting targeted tumor accumulation, and enabling controlled release. These advantages collectively contribute to improved therapeutic efficacy and reduced systemic toxicity.

Nonetheless, the development of matrine-based NDDS still faces several challenges. Issues such as potential nanocarrier toxicity, batch-to-batch variability, stability during storage, and incomplete understanding of *in vivo* behavior remain to be addressed. Moreover, the complexity of tumor microenvironments and inter-patient variability can affect the performance of NDDS, underscoring the need for more precise, adaptable, and intelligent delivery strategies (Patra et al., 2018).

Future research should focus on the rational design of matrine formulations with optimized physicochemical and pharmacokinetic properties, supported by advanced materials science and preparation technologies. The integration of artificial intelligence, machine learning, and big data analytics holds promise for accelerating formulation optimization, improving delivery precision, and supporting the development of personalized nanomedicine. Additionally, combination therapies leveraging matrine with other therapeutic agents, co-loaded into multifunctional nanocarriers, may further enhance antitumor efficacy while minimizing adverse effects.

In conclusion, while matrine-based NDDS present clear advantages and significant therapeutic potential, further systematic investigation and technological innovation are necessary to translate these findings into clinically viable cancer therapies.
